# Transcriptome Reveals Long Non-coding RNAs and mRNAs Involved in Primary Wool Follicle Induction in Carpet Sheep Fetal Skin

**DOI:** 10.3389/fphys.2018.00446

**Published:** 2018-05-15

**Authors:** Yangfan Nie, Shaomei Li, XinTing Zheng, Wenshuo Chen, Xueer Li, Zhiwei Liu, Yong Hu, Haisheng Qiao, Quanqing Qi, Quanbang Pei, Danzhuoma Cai, Mei Yu, Chunyan Mou

**Affiliations:** ^1^Key Laboratory of Agricultural Animal Genetics, Breeding and Reproduction of Ministry of Education, College of Animal Science and Technology, Huazhong Agricultural University, Wuhan, China; ^2^Qinghai Academy of Animal Science and Veterinary Medicine, Qinghai, China; ^3^Sanjiaocheng Sheep Breeding Farm, Qinghai, China; ^4^Animal Husbandry and Veterinary Station, Qinghai, China

**Keywords:** wool follicle, induction, expression pattern, skin development, long non-coding RNA

## Abstract

Murine primary hair follicle induction is driven by the communication between the mesenchyme and epithelium and mostly governed by signaling pathways including wingless-related integration site (WNT), ectodysplasin A receptor (EDAR), bone morphogenetic protein (BMP), and fibroblast growth factor (FGF), as observed in genetically modified mouse models. Sheep skin may serve as a valuable system for hair research owing to the co-existence of sweat glands with wool follicles in trunk skin and asynchronized wool follicle growth pattern similar to that of human head hair follicles. However, the mechanisms underlying wool follicle development remain largely unknown. To understand how long non-coding RNAs (lncRNAs) and mRNAs function in primary wool follicle induction in carpet wool sheep, we conducted high-throughput RNA sequencing and revealed globally altered lncRNAs (36 upregulated and 26 downregulated), mRNAs (228 elevated and 225 decreased), and 80 differentially expressed novel transcripts. Several key signals in WNT (*WNT2B* and *WNT16*), BMP (*BMP3, BMP4*, and *BMP7*), EDAR (*EDAR* and *EDARADD*), and FGF (*FGFR2* and *FGF20*) pathways, and a series of lncRNAs, including XLOC_539599, XLOC_556463, XLOC_015081, XLOC_1285606, XLOC_297809, and XLOC_764219, were shown to be potentially important for primary wool follicle induction. GO and KEGG analyses of differentially expressed mRNAs and potential targets of altered lncRNAs were both significantly enriched in morphogenesis biological processes and transforming growth factor-β, Hedgehog, and PI3K-Akt signaling, as well as focal adhesion and extracellular matrix-receptor interactions. The prediction of mRNA-mRNA and lncRNA-mRNA interaction networks further revealed transcripts potentially involved in primary wool follicle induction. The expression patterns of mRNAs and lncRNAs of interest were validated by qRT-PCR. The localization of XLOC_297809 and XLOC_764219 both in placodes and dermal condensations was detected by *in situ* hybridization, indicating important roles of lncRNAs in primary wool follicle induction and skin development. This is the first report elucidating the gene network of lncRNAs and mRNAs associated with primary wool follicle early development in carpet wool sheep and will shed new light on selective wool sheep breeding.

## Introduction

Wool, the external portion of wool follicles, is a valuable natural fiber that differs greatly in diameter, crimp, and elasticity. The refinement of wool quality, especially fiber diameter, which is highly correlated with wool follicle development, could boost the economic performance of wool sheep husbandry (Rogers, 2006). Wool follicle development includes successive generation of primary wool follicles from approximately embryonic day 55 (E55) to E65, secondary wool follicles from approximately E75 to E85, and secondary-derived wool follicles from approximately E95 to E105 in sheep fetal skin (Rogers, 2006). The fleece of carpet or coarse wool sheep is dominated by long and thick medullated wool developed from primary wool follicles, which share anatomical structures with murine primary hair follicles during early skin morphogenesis (Paus et al., 1999; Rogers, 2006).

The development of murine pelage hair follicles has been carefully classified into nine specific stages (stages 0–8) based on previously established comprehensive criteria (Paus et al., 1999). Briefly, hair follicle development progresses through *de novo* formation of the hair germ (stages 0–1), down-growth and elongation of the hair peg (stages 2–3), advanced growth of the hair peg forming an inner root sheath, outer root sheath, sebocyte, and bulge (stages 4–6), and maturation of the hair follicle (stages 7–8) across the prenatal and neonatal periods (Paus et al., 1999). The first two stages (stages 0–1) are extremely interesting for investigation, as during the progression from stage 0 to stage 1, the embryonic skin displays simple but extensive morphological changes from thin skin with homogeneous epidermal layers at stage 0 to the occurrence of periodic hair follicles (hair placode with dermal condensation beneath) and the thickening of epidermal and dermal compartments at stage 1 (Paus et al., 1999). This periodic pattern formation in skin is the outcome of molecular and cellular crosstalk between the epithelium and mesenchyme represented by wingless-related integration site (WNT), ectodysplasin A receptor (EDAR), bone morphogenetic protein (BMP), and fibroblast growth factor (FGF) signaling pathways (Paus et al., 1999; Millar, 2002; Huh et al., 2013; Glover et al., 2017). The size of hair placode and number of dermal papilla cells are highly correlated with hair fiber thickness. Hence, the periodicity and diversity of pattern formation makes hair follicle a good model to investigate the mechanisms determining organ size and shape, tissue morphogenesis, homeostasis, and stem cell dynamics. Many signature genes have been identified and characterized in the different compartments formed during this process. Of those, one study isolated specific cell types from E14.5 dorsal skin from transgenic mouse lines and identified the corresponding signature genes by RNA-seq (Sennett et al., 2015). This large-scale data combined with numerous previous reports drew a comprehensive picture of the early molecular signaling networks engaged in murine primary hair follicle morphogenesis and skin development (Millar, 2002; Schmidt-Ullrich and Paus, 2005; Rishikaysh et al., 2014).

The general principles of hair follicle development, particularly for primary hair follicles, have been widely studied in mouse models (Nakamura et al., 2013). There are advantages to manipulating mouse models for skin and hair research because of the simplicity of the dorsal skin, which lacks sweat glands as well as the clear three-wave hair follicle development and synchronized postnatal hair growth cycle (Muller-Rover et al., 2001). However, understanding of the diversity of skin and hair in humans and other mammals is limited. For example, the mechanisms underlying murine hair cycling could not fully decipher the mystery of asynchronized human head hair growth. Therefore, wool follicle may serve as a distinctive system for hair and skin study owing to its unique features. Sheep wool follicles occur and grow independently from neighboring follicles, similarly to human head hair follicles (Rogers, 2006). Sheep adult trunk skin contains both wool follicles and sweat glands, thus sharing anatomical structures with human axillary skin (Saga, 2002; Rogers, 2006). These characteristics of skin anatomy and hair growth cycling pattern in human hair follicles and wool follicles are different from those of murine skin. Hence, the investigation of sheep skin development not only adds new knowledge to understand the enigma of hair development beyond established mouse and chicken models, but also potentially provides new ideas for addressing human skin health and skincare such as human head hair loss and axillary odor or hircismus.

Long non-coding RNAs (LncRNAs) are transcripts longer than 200 nucleotides that can regulate mRNA expression at both posttranscriptional and transcriptional levels (Kornienko et al., 2013). Though several lncRNAs relevant to skin biology have been reported, such as ANCR and SPRY4-IT1 (Wan and Wang, 2014), the detailed functional roles of lncRNAs in hair and wool follicle development are based on limited reports and, thus, largely unknown. A recent study detected 15 differentially expressed lncRNAs during secondary wool follicle induction in super-fine wool sheep, which possesses no primary wool follicles due to selective breeding (Yue et al., 2016). Another study identified lncRNAs which might contribute to the variable induction ability of dermal papilla cells under tissue culture conditions (Lin et al., 2014). No studies have examined prenatal primary wool follicle development. Therefore, we assessed this earlier time point in our current report.

In this study, we focused on elucidating the global gene expression changes during the first two stages (stages 0–1) of primary wool follicle formation. The aim of this study was to explore the dynamic gene regulation during primary wool follicle induction in carpet wool sheep and improve the understanding of hair follicle and skin development. This work will provide a global illustration of the lncRNA and mRNA interaction networks during primary wool follicle induction and potentially benefit wool quality control in the wool industry.

## Materials and Methods

### Experimental Animals

Tibetan carpet sheep fetuses intended to be discarded were scavenged and immediately placed in cold PBS at a local abattoir in Qinghai Province, China. The left-sided dorsal skins were excised, cut into strips, and fixed in 4% paraformaldehyde at 4°C for approximately one week prior to hematoxylin and eosin (H&E) staining. The right-sided dorsal skins were chopped into pieces and frozen in liquid nitrogen for RNA extraction. More than 60 individuals at unspecified embryonic stages were randomly collected and predicted to range approximately from E45 to E75 roughly based on the body size and length. The accurate developmental stages of wool follicles in sheep skin were determined by H&E staining in the next step. All experiments on animals were approved by the Standing Committee of Hubei People’s Congress and the ethics committee of Huazhong Agricultural University.

### Morphological Identification of Primary Wool Follicle Induction in Carpet Wool Sheep

To identify the developmental stages of wool follicles, more than 60 (*n* ≥ 60) fixed sheep skin samples were dehydrated and processed in paraffin, according to the standard procedures. H&E staining was conducted using 5-μm skin sections. The stained skin sections were photographed individually and carefully grouped into different developmental stages based on previous reports (Paus et al., 1999). The induction stages of primary wool follicles were further investigated. Six individuals comparable to E55 and E65, corresponding to stages 0 and 1 of primary wool follicle morphogenesis were selected for RNA isolation and high throughput sequencing, respectively (*n* = 3).

### RNA Extraction and cDNA Synthesis

Total RNA was extracted using TRIzol Reagent (Invitrogen, United StatesUSA) and processed for quality determination using a NanoDrop 2000 spectrophotometer (Thermo Scientific, United States). The cDNA was generated for qRT-PCR validation using the PrimeScript^TM^ RT reagent Kit with gDNA Eraser (Takara, Japan).

### Illumina Sequencing and Data Analysis

Six libraries of sheep skin samples were constructed and sequenced on the Illumina HiSeq 2000 (San Diego, CA, United States) with 150 base pair paired-end reads at Novogene (Beijing, China) following the manufacturer’s procedures. Briefly, total RNA was extracted using TRIzol reagent, and RNA integrity was assessed using the RNA Nano 6000 Assay Kit with the Bioanalyzer 2100 system (Agilent Technologies, United States). The sequencing libraries were prepared from total RNA devoid of ribosomal RNA using the NEBNext^®^ Ultra^TM^ Directional RNA Library Prep Kit for Illumina^®^ (NEB, United States). The products were purified with an AMPure XP system (Beckman Coulter, United States) and assessed on an Agilent Bioanalyzer 2100 system for quality control. After cluster generation, the libraries were sequenced. All reads were deposited in the NCBI Sequence Read Archive (SRA) database under accession number SRP126454. Clean reads were mapped to the sheep reference genome (version: Oarv3.1). Transcripts without coding potential, predicted by Coding-Non-Coding Index (CNCI) (v2) (Sun et al., 2013), Coding Potential Calculator (CPC) (0.9-r2) (Kong et al., 2007), Pfam-scan (v1.3) (Punta et al., 2012), and Phylogenetic codon substitution frequency (phyloCSF) (v20121028) (Lin et al., 2011), were candidate lncRNAs. The conservation of lncRNAs and mRNAs was analyzed using Phast (v1.3) software (Siepel et al., 2005). Coding genes 10 k/100 k upstream and downstream of lncRNAs were predicted as target genes in *cis*, while targets in *trans* were predicted via calculating the expressed correlation with lncRNAs. For biological replicates, lncRNAs and mRNAs with *p*-adjust <0.05 were regarded as differentially expressed between the two stages of primary wool follicle induction. GOseq R packages (Young et al., 2010) and KOBAS software (Mao et al., 2005) were used for Gene Ontology (GO) and KEGG analysis of the differentially expressed lncRNA targets and mRNAs, respectively.

### Interaction Network Construction

Protein-protein interaction analysis of differentially expressed genes was based on the commonly used STRING database. Due to the absence of sheep in this database and limited studies on skin and hair development of the relative species Bos taurus, we used the mouse genome for the interaction network construction. Briefly, the sequences of differentially expressed lncRNA targets and mRNAs were blasted (blastx) to the *Mus musculus* genome to predict protein-protein interactions using the STRING database^1^. Then, the mRNA-mRNA and lncRNA–mRNA interaction networks were visualized in Cytoscape (Shannon et al., 2003).

### Quantitative Real-Time PCR (qRT-PCR) Validation

Several differentially expressed mRNAs and lncRNAs involved in primary wool follicle induction were selected and confirmed by qRT-PCR with *GAPDH* used as an internal reference. The primers for qRT-PCR are listed in **Table 1**. qRT-PCR was carried out with a Roche LightCycler^®^ 96 using iTaq^TM^ Universal SYBR^®^ Green Supermix (Bio-Rad, United States). The amplification procedures were 95°C for 5 min initially, followed by 45 cycles of 95°C for 15 s and 60°C for 1 min. Quantification of mRNAs and lncRNAs was performed using the standard curve method with average cycle thresholds (Ct). The qRT-PCR data were generated from six independent samples per stage, including the three original samples subjected to RNA sequencing and three additional samples, and statistically analyzed using Student’s *t*-test (*n* = 6).

**Table 1:** The list of primers for qRT-PCR.

Genes and LncRNAs	Forward (5′–3′)	Reverse (5′–3′)
EDAR	TGCTATCGCTGGTCCACCT	ACCGCCTTCTCCGAGTTGTA
EDARADD	AAGGAACCAGTGGAAGACACAGAC	AGCGAACAGGAGGGACAGGAA
FOXI3	ACGCTCAGCCACATCTACCAGTTC	TCCAGGGTCCAGTAATTGCCTTTCC
SHH	TGAACGCCTTAGCCATCTCC	GGTCCGAGGTGGTGATGTC
SOSTDC1	CAGCAGCAACAGCACAATGA	ATCCAGTTAGGCAGCACAGG
BMP3	GCAGATATTGGCTGGAGCGAATG	AAGAACAGGATGCTGAGTGAGGAC
BMP4	CACCACGAAGAACATCTGGAGAACA	CGGCAGACGAGATCACCTCATTC
BMP7	GACCAGAGGCAGGCATGTAAGAAG	CTCACAGTAGTAGGCGGCATAGC
WNT16	CGAGTGAAGGCTGGCATTG	CGGCAGTCTAAGGACATCAAC
WIF1	ACCGTCAATGTCCCTCTTCTG	TGCTGCCACTCCATCTTGTT
LEF1	CAGGTGGTGTTGGACAGATAAC	GACAGTGAGGATGGGTAGGG
SMOC1	AGAGCAAGTGTCGCCTGGAG	CACAGAAGAGCCGCTGATGG
GAPDH	GCGACACTCACTCTTCTACCTTC	TCTCTTCCTCTCGTGCTCCTG
XLOC_539599	GGACGCAGTGGCATCATTC	CCGAGAGTCTCACAACAAACC
XLOC_556463	CCGAGACTAACCTTGGATTGG	CTCATTCAGTGTGTCAGATGTTTC
XLOC_015081	AAGAGGTGGTGTGGAGTAACAG	GAGCCAGCAAGCAGCAAAG
XLOC_1285606	CAGGTGACCAGGGACATCTC	TGCTGTGGAATGGCTGCTT
XLOC_297809	ACCGACACAGCCGTAAAGC	TGGAGTCCATGCGTCTAAGC
XLOC_764219	GGTCCGTTGGGTATCCTCTG	CCTGCCACTCTTGACTCCTT

### *In Situ* Hybridization

Antisense riboprobes were generated and labeled with Digoxigenin (Roche, United States) by *in vitro* transcription using linearized plasmids as templates, and then applied to 10-μm sheep skin sections for *in situ* hybridization as described previously with minor modifications (Lee et al., 2013). Briefly, the skin sections were dewaxed and rehydrated to process with proteinase K treatment. After a 15 min fixation in 4% paraformaldehyde and pre-hybridization, the sections were hybridized overnight at 58°C with riboprobes in the hybridization solution. The sections were rinsed in gradient SSC as descried previously (Lee et al., 2013). Then the sections were washed with TBST buffer for 10 min and incubated with 10% BSA, followed by incubation with Anti-Digoxigenin-AP Fab fragments (Roche, United States, 1:1000) overnight. The sections were washed with TBST buffer and incubated with the BCIP/NBT (Sigma, United States) solution to detect hybridized signal. The hybridized signals were photographed by microscopy (Olympus BX53, Tokyo, Japan). Corresponding sense riboprobes were generated and applied to the skin sections for *in situ* hybridization as negative controls.

### Statistical Analyses

All data are presented as the mean ± SEM. All statistical tests were performed using GraphPad Prism version 6 software (United States). The Student’s *t*-test was used to assess the differences between two groups. Statistically significant differences were determined at a *p*-value < 0.05.

## Results

### Morphological Characterization of Primary Wool Follicle Induction in Carpet Wool Sheep Skin

**FIGURE 1:**
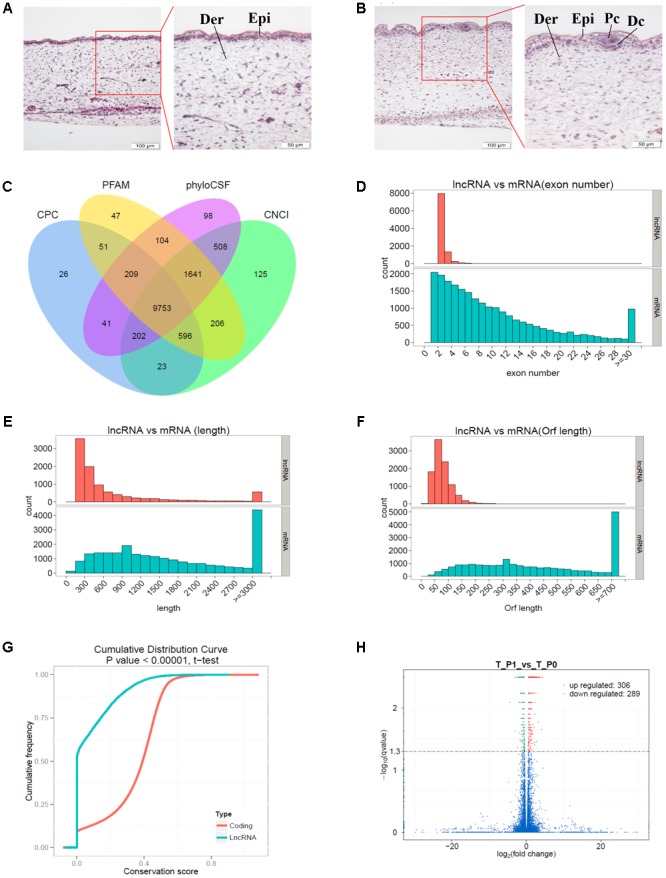
Identification of long non-coding RNAs (lncRNAs) and mRNAs involved in primary wool follicle induction stages in carpet wool sheep fetal skins. **(A,B)** The morphology of skin and wool follicles at stages 0 and 1 of primary wool follicle development were observed by hematoxylin and eosin (H&E) staining **(Left)** are 200×, scale bars represent 100 μm. **(Right)** are 400×, scale bars represent 50 μm, which is the magnification of the segment in the red frame). Epi, epidermis, Der, dermis, Pc, placode, Dc, dermal condensation. At stage 1 **(B)**, the hair placode and dermal condensation were clearly detected compared to the homogeneous epidermal layers at stage **(A)**. **(C)** Coding potentiality filter using Coding Potential Calculator, Pfam, Phylogenetic codon substitution frequency, and Coding-Non-Coding Index. **(D)** Exon number distribution of lncRNAs and mRNAs. **(E)** Transcript lengths distribution of lncRNAs and mRNAs. **(F)** Open reading frame length distribution of lncRNAs and mRNAs. **(G)** Conservation of the sequence in lncRNAs and mRNAs was analyzed using Phast (v1.3) software. **(H)** Differentially expressed transcripts in sheep skin between the two stages of primary wool follicle development. Red, green, and blue dots in the graph represent transcripts that were significantly upregulated, significantly downregulated, and not significantly changed between these two stages, respectively.

The dorsal skin samples obtained from Tibetan carpet wool sheep fetuses (*n* ≥ 60) were randomly collected and grouped to identify the developmental stages of primary wool follicles by H&E staining (**Figures 1A,B**). The development of skin and wool follicles was detected across early and late stages, based on a series of H&E stained skin sections. The specific induction stages, stage 0 and stage 1 of the primary wool follicle development, which correspond to approximately embryonic day E55 and E65, respectively, were deliberately picked for further classification. At stage 0, the epidermis of the sheep fetal skin was observed thin and uniform, whereas at stage 1, it thickened dramatically with regularly spaced wool placodes attached with initiated dermal condensations. The dermis of the sheep skin was also thickened compared to that at stage 0. These characteristics were utilized to carefully classify wool follicles in sheep skin into developmental stage 0 and stage 1. (**Figures 1A,B**). More than six individuals in each group were further examined.

### Identification of LncRNAs and mRNAs in Carpet Wool Sheep Fetal Skin

Illumina sequencing of six cDNA libraries derived from carpet wool sheep whole skin yielded a total of 338,366,742 and 305,668,486 raw reads for stage 0 and stage 1 samples, respectively. Clean reads devoid of adapter and low-quality sequences occupied more than 96% of the raw data (**Table 2**). Nearly 81.15% of the clean reads were mapped to the sheep reference genome, of which more than 74.44% were uniquely mapped.

**Table 2:** Statistics of clean reads in carpet wool sheep fetal skin.

Sample name	Raw reads	Clean reads	Clean bases	Total mapped	Multiple mapped	Uniquely mapped
T_P0_1	154814210	149258426 (96.41%)	22.39G	117986653 (79.05%)	4911394 (3.29%)	113075259 (75.76%)
T_P0_2	88743300	86003912 (96.91%)	12.9G	68722034 (79.91%)	3670783 (4.27%)	65051251 (75.64%)
T_P0_3	94809232	91320540 (96.32%)	13.7G	70867990 (77.60%)	2893463 (3.17%)	67974527 (74.44%)
T_P1_1	102503068	100452360 (98.0%)	15.07G	80046354 (79.69%)	3406082 (3.39%)	76640272 (76.30%)
T_P1_2	106002884	103889830 (98.0%)	15.58G	84304287 (81.15%)	4118722 (3.96%)	80185565 (77.18%)
T_P1_3	97162534	95292614 (98.08%)	14.29G	75539233 (79.27%)	3444940 (3.62%)	72094293 (75.66%)

A total of 9,753 lncRNAs were identified after coding potential filter using CNCI, CPC, Pfam-scan, and phyloCSF software (**Figure 1C**). In addition, 22,800 mRNAs and 3,975 novel transcripts were identified. The exon number, transcript length, and ORF length of lncRNAs and mRNAs were calculated and graphed, as shown in **Figures 1D–F**. A majority of lncRNAs comprised two or three exons, whereas mRNAs contained a broad range of exon numbers from two to thirty (**Figure 1D**). The transcript length (**Figure 1E**) and ORF length (**Figure 1F**) of lncRNAs were significantly shorter than those of mRNAs. Moreover, most lncRNAs were less conserved than mRNAs (**Figure 1G**).

### Functional Annotation of Differentially Expressed LncRNAs and mRNAs in Primary Wool Follicle Induction

During the early development of wool sheep fetus, anatomical changes were observed in trunk skin between stages 0 and 1 of primary wool follicle development (**Figures 1A,B**). The most prominent structures were the appearance of the follicle placode and dermal condensation (**Figures 1A,B**). RNA sequencing analyses detected 595 differentially expressed transcripts between these two stages (**Figure 1H**). Of these, there were 36 upregulated and 26 downregulated lncRNAs, together with 228 upregulated and 225 downregulated mRNAs (**Supplementary Table S1**). Additionally, 80 differentially expressed novel transcripts were obtained.

These differentially expressed transcripts were divided into four classes based on the structure changes of sheep embryonic skin during primary wool follicle induction (**Figures 1A,B**). The established hair follicle marker genes, including placode markers (*EDAR, FOXI3, FGF20, SHH*, and *SOSTDC1*), dermal condensation markers (*TRPS1, CXCR4, BMP3, BMP4, BMP7*, and *TBX18*), epidermal markers (*KRT1, KRT10, DSC1, DMKN*, and *SLC7A11*), and fibroblast markers (*DPP4* and *LOX*) showed elevated expression at stage 1 when primary wool follicles were initiated (Sennett et al., 2015; Rezza et al., 2016). Further analyses revealed that the key signaling pathways regulating murine primary hair follicle induction, including WNT, BMP, EDAR, and FGF, were detected in our data. For example, several genes in the WNT signaling pathway (*WNT16, LEF1*, and *WIF1*) were significantly upregulated at stage 1. Three BMPs, including *BMP3, BMP4*, and *BMP7*, showed elevated expression in stage 1 samples. *SOSTDC1*, a modulator of both the WNT and BMP pathways, displayed increased expression at stage 1 in sheep skin. In addition, several components of the EDAR signaling pathway (*EDAR, EDARADD*, and the downstream effector *FOXI3*) were highly enriched at wool follicle induction stage 1.

**FIGURE 2:**
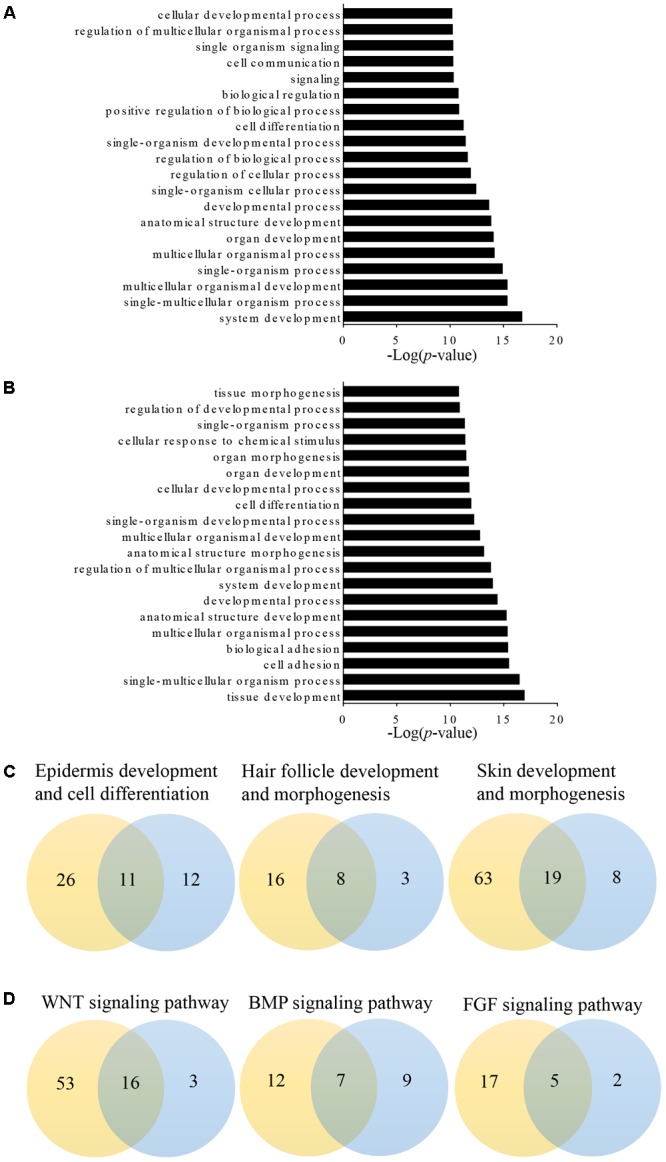
Gene Ontology (GO) analysis of differentially expressed lncRNAs targets and mRNAs in primary wool follicle induction. **(A,B)** The top 20 enrichment biological processes for differentially expressed lncRNA targets and mRNAs are listed. **(C)** One-to-one pairs of differentially expressed lncRNA targets and mRNAs involved in wool follicle and skin development. **(D)** One-to-one pairs of differentially expressed lncRNA targets and mRNAs involved in key signaling pathways in wool follicle development. The orange and blue circles represent the number of differentially expressed lncRNA targets and mRNAs, respectively. Their intersections represent the number of differentially expressed targets of differentially expressed lncRNAs.

Long non-coding RNAs may regulate gene expression by binding and modulating the activity and stability of the target genes. A total of 7,677 targets of 62 differentially expressed lncRNAs were predicted and classified based on the *cis* and *trans* principles (**Supplementary Table S2**). GO terms of these lncRNA potential targets were highly enriched for several developmental processes, such as system, organ, anatomical structure, multicellular organismal, and cellular development (**Figure 2A** and **Supplementary Table S3**). The predicted lncRNA targets partially overlapped with the differentially expressed mRNAs, which were highly enriched for morphogenesis biological processes, such as system development, anatomical structure development and morphogenesis, and organ development and morphogenesis (**Figure 2B** and **Supplementary Table S4**). Of these, 11, 8, and 19 overlapped target genes were identified and predicted to function in epidermal development and cell differentiation, hair follicle development, and skin development and morphogenesis, respectively (**Figure 2C** and **Table 3**). GO analyses revealed signaling pathways important in hair follicle development, including WNT, BMP, and FGF, which shared 16, 7, and 5 genes with lncRNA targets, respectively (**Figure 2D** and **Table 3**).

**Table 3:** Summary of differentially expressed long non-coding RNA (lncRNA) targets and mRNAs involved in primary wool follicle induction and skin development.

Epidermis development, morphogenesis, and cell differentiation	lncRNA targets	ADAM9 ALX4 **BMP4** CD109 CLRN1 DLL1 **EDARADD FGFR2** FOXN1 H2AFY2 **HPSE KCNMA1** KDF1 KIF3A KRT36 KRT84 LGR5 MAP2K1 MCOLN3 NAB1 NAB2 NOTCH1 OVOL2 PALLD PPARA **PTCH1** ROCK1 ROCK2 **SFRP4 SHH SOSTDC1** SOX21 **TFAP2C** TMC1 **TP63** TXNIP VDR
	
	mRNA	**BMP4** CDH3 CYP26B1 EDAR **EDARADD FGFR2** GLI1 GRHL2 **HPSE** IGFBP5 **KCNMA1** KRT10 LRP4 POU2F3 **PTCH1 SFRP4 SHH** SLITRK6 **SOSTDC1 TFAP2C** TGM3 **TP63** WNT16

Hair follicle development and morphogenesis	lncRNA targets	ALX4 APC APCDD1 ATP7A CD109 CELSR1 CTSV **EDARADD FGFR2** FOXN1 GNAS **HPSE** LDB1 LGR5 LHX2 **LRP4** NOTCH1 **SHH** SNAI1 **SOSTDC1** SOX21 SOX9 **TFAP2C TP63**
	
	mRNA	CDH3 EDAR **EDARADD FGFR2 HPSE** IGFBP5 **LRP4 SHH SOSTDC1 TFAP2C TP63**

Skin development and morphogenesis	lncRNA targets	ABCA12 ABCB6 ADAM9 ADAMTS2 ALOX12B ALX4 APC APCDD1 ATP7A **BMP4** CASP3 CD109 CDSN CELSR1 CLRN1 COL1A2 CTSV CUX1 **CYP26B1** DHCR24 DLL1 **EDARADD** EPHA2 ERCC3 **FGFR2** FOXN1 **GLI1** GLI2 GNAS GRXCR1 H2AFY2 HES5 **HPSE** IFT172 **ITGA3 ITGB4 KCNMA1** KDF1 KIF3A KLF4 KRT36 KRT84 KRT9 LDB1 LGR5 LHFPL5 LHX2 **LRP4** MAP2K1 MCOLN3 MYO6 MYO7A NAB1 NAB2 NOTCH1 OVOL2 PALLD PAX6 **POU2F3** PPARA **PTCH1** ROCK1 ROCK2 **SFRP4 SHH SLITRK6** SNAI1 **SOSTDC1** SOX21 SOX9 TCF15 TCF7L2 **TFAP2C** TGM1 TMC1 **TP63** TXNIP USH2A VDR WDPCP **WNT16** WNT5A
	
	mRNA	**BMP4** CDH3 **CYP26B1** EDAR **EDARADD FGFR2 GLI1** GRHL2 **HPSE** IGFBP5 **ITGA3 ITGB4 KCNMA1** KRT1 KRT10 **LRP4 POU2F3 PTCH1 SFRP4 SHH SLITRK6 SOSTDC1** TFAP2B **TFAP2C** TGM3 **TP63 WNT16**

WNT receptor signaling pathway	lncRNA targets	APC ARNTL ASPM BARX1 BICC1 CDC73 **CDK14** CELSR1 DD1 DISC1 DLX5 DRAXIN FERMT2 **FGFR2** FGFR3 FRZB FZD2 FZD4 FZD5 **GATA3 GLI1** GSC HIC1 ILK **ITGA3** KLF4 LDB1 LGR5 LGR6 **LRP4** MAD2L2 MAPK14 MBD2 MCC MDFI MKS1 MLLT3 NFKB1 NKD2 NOG NOTCH1 NPHP3 **PITX2** PPAP2B **PTPRO** RNF43 RU RYK **SFRP1 SFRP4 SHH** SIAH2 **SOSTDC1** SOX2 **SOX4** SOX9 **SULF1** TCF7L2 TDGF1 TLE4 TNKS UBR5 USP34 **WNT16 WNT2B** WNT5A ZBED2 ZEB2 ZNF703
	
	mRNA	CDH3 **CDK14 FGFR2 GATA3 GLI1 ITGA3** LEF1 **LRP4** MYOC **PITX2 PTPRO SFRP1 SFRP4 SHH SOSTDC1 SOX4 SULF1 WNT16 WNT2B**

BMP signaling pathway	lncRNA targets	**BMP4** CHRDL2 **CYR61** FKBP8 HES5 HTRA1 HTRA3 ILK **ITGA3** NOG NOTCH1 **RGMA SFRP1 SOSTDC1 SULF1** TCF7L2 TOB1 TRIM33 WNT5A
	
	mRNA	ACVR2A **BMP4** BMP7 BMPER COL2A1 **CYR61** GATA3 ID1 **ITGA3** LEF1 LRP4 **RGMA SFRP1 SOSTDC1 SULF1** TFAP2B

FGF receptor signaling pathway	lncRNA targets	DSTYK EGR3 FGF17 FGF19 FGF23 **FGFR2** FGFR3 FRS2 **GATA3** HHIP KLB LRIT3 NDST1 NOG **SFRP1** SPATA5 SPRY1 **SULF1** TCF7L2 TDGF1 **THBS1** WNT5A
	
	mRNA	FGF20 **FGFR2 GATA3 SFRP1** SPRY2 **SULF1 THBS1**

*Genes marked in bold indicate the potential lncRNA targets that overlapped with mRNAs in the specified primary wool follicle induction stage.*

**FIGURE 3:**
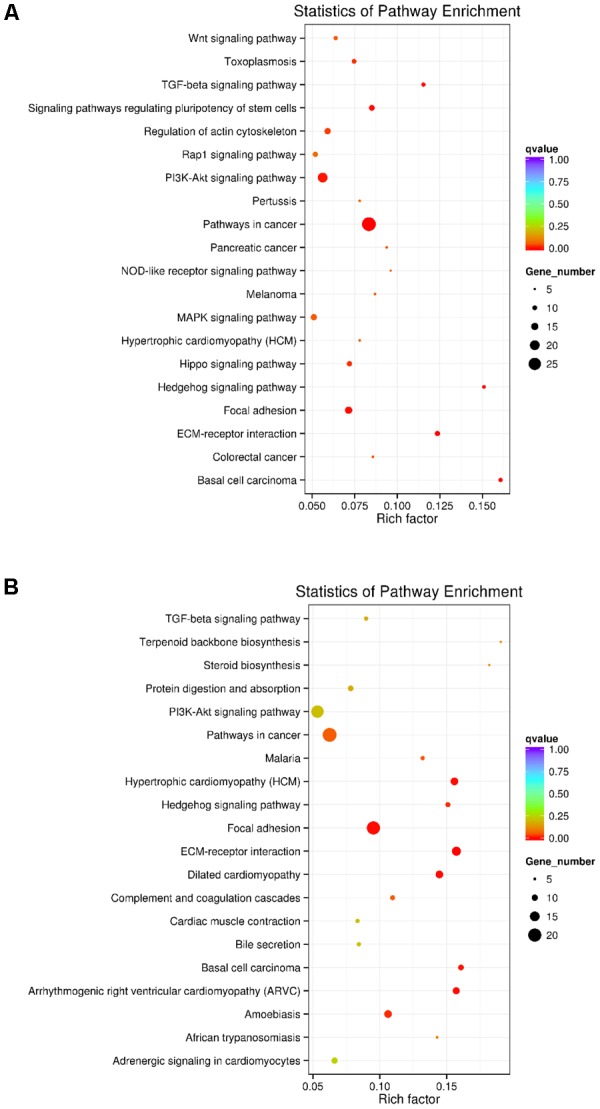
KEGG analysis of differentially expressed lncRNAs targets and mRNAs in primary wool follicle induction. **(A,B)** The top 20 KEGG enrichment pathways for differentially expressed lncRNA targets and mRNAs are presented. The longitudinal and horizontal axis represents the enrichment pathways and rich factor of these pathways, respectively. Spot size represents the number of differentially expressed genes enriched in each pathway, and the color of the spot represents the *q*-value of each pathway.

KEGG analyses revealed that these differentially expressed lncRNA target genes related to hair follicle and skin development were enriched in 32 significant terms, such as WNT, TGF-β, Hedgehog, and PI3K-Akt signaling pathways, focal adhesion, ECM-receptor interaction, and signaling pathways regulating pluripotency of stem cells (**Figure 3A** and **Supplementary Table S5**). KEGG analyses of differentially expressed mRNAs were also enriched in four of the five pathways mentioned above except for the WNT signaling pathway (**Figure 3B** and **Supplementary Table S6**).

### mRNA–mRNA and LncRNA–mRNA Interaction Network in Primary Wool Follicle Induction

**FIGURE 4:**
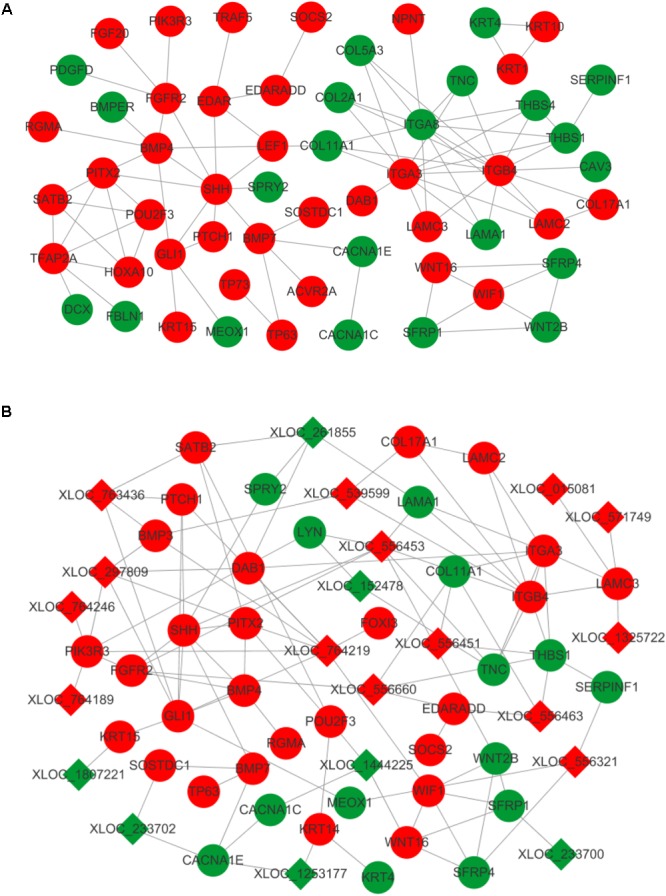
Interaction network in primary wool follicle induction. **(A)** mRNA–mRNA interaction network involved in hair follicle development was constructed and is presented. **(B)** lncRNA–mRNA interaction network related to hair follicle development is displayed. Red and green represent upregulated and downregulated, respectively. Circles and squares represent mRNAs and lncRNAs, respectively.

A number of transcripts related to hair and skin development were applied to construct mRNA–mRNA and lncRNA–mRNA interaction networks. Of these, differentially expressed mRNAs were grouped and found to regulate the proliferation and differentiation of epidermal keratinocytes (*KRT1, KRT4, KRT10, KRT15*, and *TP63*), dermal-epidermal junction and basement membrane (*COL17A1, LAMA1, LAMC2, LAMC3, ITGA3, ITGA8*, and *ITGB4*), and dermal compartment (*COL5A3* and *COL2A1*) (Pulkkinen et al., 1994; Kivirikko et al., 1995; McGrath et al., 1995; Mills et al., 1999; Imamura et al., 2000; Natsuga et al., 2011; Has et al., 2012; Talbot et al., 2016). Transcripts regulating hair placode and dermal condensation were also enriched and represented by the WNT (*WNT2B, WNT16, SFRP1, SFRP4, SERPINF1, LEF1*, and *WIF1*), EDAR (*EDAR, EDARADD*, and *TRAF5*), BMP (*BMP4, BMP7, BMPER, ACVR2A, RGMA*, and *SOSTDC1*), SHH (*SHH, PTCH1*, and *GLI1*), and FGF (*FGFR2* and *FGF20*) signaling pathways (**Figure 4A**).

The altered lncRNAs were also highly correlated with development of the epidermis, dermis, dermal condensation, and wool placode. The elevated expression of lncRNAs was predicted to target laminins (*LAMC3*), collagens (*COL17A1*), WNT signals (*WIF1* and *SFRP4*), BMP signals (*BMP3* and *BMP4*), SHH signals (*SHH, PTCH1*, and *GLI1*), and EDAR signals (*EDARADD* and *FOXI3*). This group of lncRNAs may regulate epidermis, dermis, and placode formation through their targets. The downregulated lncRNAs were predicted to interact with keratins (*KRT14* and *KRT15*), BMP signals (*SOSTDC1*), WNT signals (*WNT16* and *SFRP1*), and laminins (*LAMA1*), to mainly influence epidermal and wool placode development. These results demonstrate a complex regulatory relationship between lncRNAs and mRNAs in primary wool follicle induction (**Figure 4B**).

### Validation of Potential mRNAs and LncRNAs Functioning in Primary Wool Follicle Induction by qRT-PCR and *in Situ* Hybridization

**FIGURE 5:**
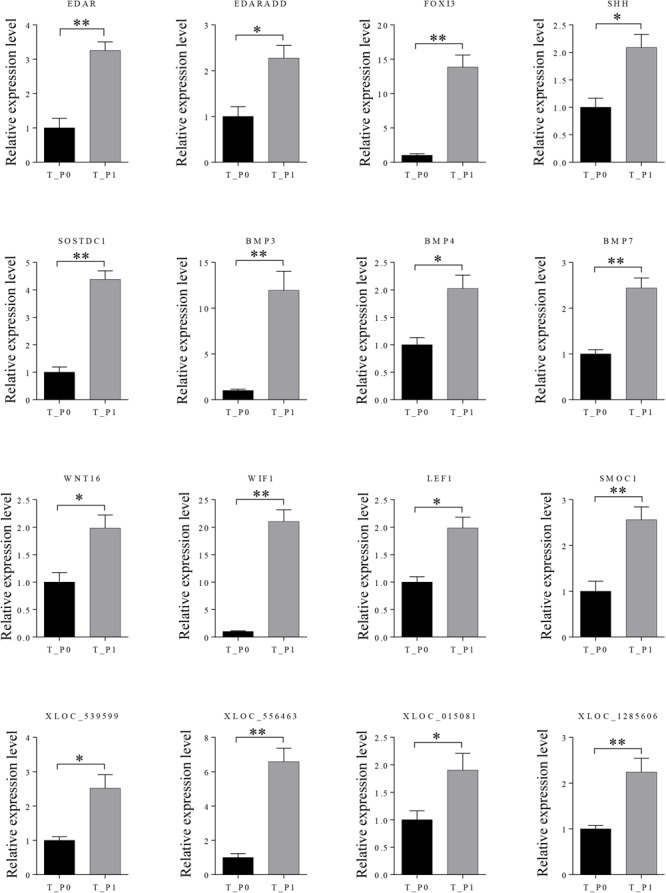
Expression levels of differently expressed mRNAs and lncRNAs involved in primary wool follicle induction were validated by qRT-PCR. Data are presented as mean ± SEM (*n* = 6). ^∗^*p* < 0.05, ^∗∗^*p* < 0.01 (Student’s *t*-test).

Twelve mRNAs highly related to wool placode and dermal condensation formation (*EDAR, EDARADD, FOXI3, SHH, SOSTDC1, BMP3, BMP4, BMP7, WNT16, WIF1, LEF1*, and *SMOC1*) and four lncRNAs (XLOC_539599, XLOC_556463, XLOC_015081, and XLOC_1285606) possibly essential for primary wool follicle induction were selected and validated by qRT-PCR at the first two induction stages in wool follicle development. The qRT-PCR results of these mRNAs and lncRNAs were consistent with those from the sequencing data (**Figure 5**).

**FIGURE 6:**
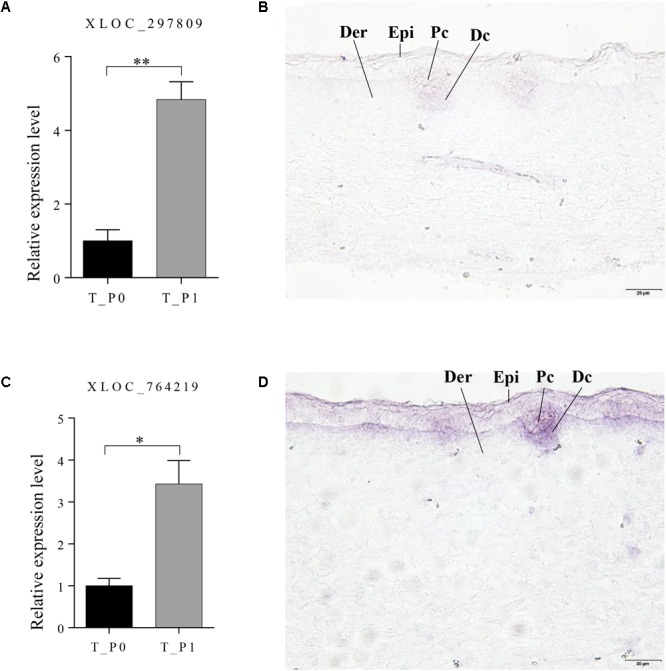
Expression pattern of XLOC_297809 and XLOC_764219 at stage 1 of primary wool follicle induction. **(A,B)** qRT-PCR and *in situ* hybridization of XLOC_297809. XLOC_297809 was expressed in placodes and dermal condensations. **(C,D)** qRT-PCR and *in situ* hybridization of XLOC_764219. XLOC_764219 was expressed in epidermis, placodes, and dermal condensations. Data are presented as the mean ± SEM (*n* = 6). ^∗^*p* < 0.05, ^∗∗^*p* < 0.01 (Student’s *t*-test). Skin sections at stage 1 of primary wool follicle development in sheep fetus were displayed at 400× (scale bars represent 20 μm). Epi, epidermis, Der, dermis, Pc, placode, Dc, dermal condensation.

Two lncRNAs (XLOC_297809 and XLOC_764219), which potentially target *BMP3* and *FOXI3*, were chosen to detect the expression patterns at stage 1 in primary wool follicle induction by *in situ* hybridization. Both lncRNAs were detected in wool placode and dermal condensation. XLOC_297809 was restricted in the placode and dermal condensation, whereas positive XLOC_764219 signals were strong in these two structures, but weaker in the epidermal layers (**Figure 6**). In negative controls hybridized with corresponding sense riboprobes, no hybridized signal was detected in the skin sections with either the XLOC_297809 or XLOC_764219 probe (**Supplementary Figure S1**).

## Discussion

Carpet wool sheep are characterized by long and thick wool fiber which is developed from primary wool follicles. In sheep skin, the primordia of primary follicles develop in a trio pattern defined by one central and two lateral follicles and finally organize in a hexagonal pattern (Rogers, 2006). This occurs between E55 and E65 (Ruttle and Sorensen, 1965; du Cros et al., 1992; Wynn et al., 1995; Xavier et al., 2013), which is comparable to the developmental stages E13.5 to E14.5 of primary hair follicle in murine dorsal skin (Millar, 2002; Schmidt-Ullrich and Paus, 2005). During these particular primary follicle induction stages, murine and sheep skin share complicated anatomical changes including *de novo* formation of the wool placode and dermal condensation, as well as thickening of the epidermis and dermis, which are marked with different signature genes. The similar morphological changes and different duration of induction periods indicate that the mechanism regulating wool and hair primary follicle induction could be mostly conserved with subtle differences. It may also explain the differences observed in our data compared to mouse data. A previous RNA sequencing analysis identified 102, 395, and 254 signature genes specific for development of the placode, dermal condensation, and epidermis in E14.5 murine skin, respectively (Sennett et al., 2015). Of these, only 14, 29, and 19 genes were differentially expressed in the wool placode, dermal condensation, and epidermis in our data, respectively (**Table 4**). The remaining genes did not show significantly altered expression. Interestingly, a total of 27, 90, and 66 genes specific for these three structures, respectively, were not found in our data. This could be partially explained that the murine data showed slight differences among the different skin compartments at the hair placode stage, while our data generated from the whole skin compared gene regulation between two stages (Sennett et al., 2015). Another explanation might be due to some genes being restricted by expressing location without changing the expression levels. These enriched mRNAs in our data revealed that primary wool follicle induction shares a number of key genes driving morphological changes as those in murine primary hair follicles.

**Table 4:** List of significantly expressed signature genes in placode, dermal condensation, and epidermis.

Signature gene location	Signature gene name
Placode	BNC1 CDH3 EDAR FGF20 FGFR2 FOXI3 FREM2 FRY LAMC2 LRP4 RASGEF1B SHH SOSTDC1 SUSD4
Dermal condensation	ANO2 APOD ARL15 BMP3 BMP4 BMP7 CADPS2 CCDC3 COL7A1 CXCR4 CYP26B1 FREM1 GLI1 LAMC3 NDNF NPTX2 PDLIM3 PRDM1 PTPRO S100A4 SMOC1 SPOCK1 SPON2 TBX18 TNC TMEM35 TRPS1 WFIKKN2 WIF1
Epidermis	DMKN DPP4 DSC1 DSC2 DSC3 F2RL1 FAM84A KRT1 KRT10 KRT23 KRT4 PIK3C2G PLA2G4F RAPGEFL1 SERPINB12 SLC7A11 SPTLC3 TGM3 THEM5

The mRNA–mRNA interaction network showed two complex systems responsible for follicle placode and dermal condensation as well as epidermal differentiation and homeostasis, implying conserved signals functioned in both primary hair follicle and primary wool follicle induction. At the earliest stage of primary hair follicle morphogenesis, the communication between the epithelium and mesenchyme drives formation of the placode, dermal condensation niche, and thickened epidermis and dermis, which requires a cascade of gene networks including WNT, EDAR, BMP, and FGF signaling pathways (Paus et al., 1999; Millar, 2002; Huh et al., 2013; Glover et al., 2017).

Canonical WNT/β-catenin signaling is essential for hair follicle induction, as ectopically expressed WNT inhibitor Dickkopf 1 (*Dkk1*) or specifically knocked-out β-catenin in epidermis both resulted complete blockage of hair follicle induction (Huelsken et al., 2001; Andl et al., 2002). Two WNTs (*WNT2B* and *WNT16*) and other signaling molecules in WNT (*SFRP1, SFRP4, LRP4, LEF1*, and *WIF1*) were significantly enriched in our data, suggesting crucial roles of the WNT signaling pathway in primary wool follicle induction. *Wnt2* was mainly expressed in mouse primary hair placode (Fu and Hsu, 2013). *WNT2B*, also known as *WNT13*, was significantly downregulated by approximately twofold at stage 1 in our data, which shares similar expression trend with *WNT2* during the induction of secondary wool follicle development, as reported previously (Yue et al., 2016). The expression of *WNT2B* was stimulated in human keratinocytes following *BMP2* application (Yang et al., 2006), indicating that *WNT2B* might act as a potential BMP target in skin and hair follicle development. In our data, *WNT16* was upregulated by approximately twofold during primary wool follicle induction and may be regulated by XLOC_1444225. *Wnt16* was mostly expressed in the inter-follicular epithelium, suggesting that it restricts the hair placode edge in the placode stage (Reddy et al., 2001). However, its actual function in early development has not been examined in detail, although it may function in hair follicle bulge stem cell regulation (Kandyba et al., 2013). WNT inhibitory factor 1 (*Wif1*) is a marker of dermal condensation that displays a spot-like expression pattern in the developing murine whisker follicles (Hsieh et al., 1999). *WIF1*, a potential target of XLOC_764219 in our data, was upregulated at the placode stage, indicating its importance in primary wool follicle dermal condensation formation. Unexpectedly, both *WNT10A* and *WNT10B*, which are important in early hair follicle formation (Reddy et al., 2001; Andl et al., 2002; Zhang et al., 2009), did not show altered expression in our sequencing data. Whether their expression location is changed rather than their expression levers requires further elucidation.

The activation of WNT/β-catenin signaling drives the homogenous epidermis to become competent and gain follicular or inter-follicular cellular fates forming rudimentary hair follicle placodes (Zhang et al., 2008). These follicle precursors need other signals to reinforce and shape the pre-pattern to finally establish the dense and clear-edged hair follicle placode. Our previous studies together with other reports confirmed that *Edar* functions as a key activator negotiating with the inhibitor, *Bmp*, to refine the pre-pattern into fine-patterned, well-organized hair follicle placode (Headon and Overbeek, 1999; Botchkarev et al., 2002; Mou et al., 2006). Extensive studies showed that the receptor Edar, not its ligand Eda, is a key stimulator for primary hair follicle induction, as overexpression of *Edar* in the epidermis could rescue primary hair follicle formation in a cross of *Edar* transgenic mice with *Eda* mutant mice (Mou et al., 2008). The elevated expression of *EDAR* and its intracellular adaptor *EDARADD* indicates their important roles in wool placode induction. *EDA* did not show convincing expression change supporting the notion that *EDA* is not a key signal during the induction stage. *Foxi3*, a potential target of XLOC_764219, is a downstream effector of *Eda* in hair placode formation. However, unlike other molecules involved in EDA/EDAR/NF-kB signaling, *Foxi3* knockout mice have normal primary hair placodes (Shirokova et al., 2013, 2016). Mostly likely, *EDA*, and *FOXI3* are not key signals that stimulate primary wool follicle induction. These results suggest that the EDAR signaling pathway is important for primary wool follicle induction. It would be interesting to explore sheep *EDAR* function in later developmental stages.

*Edar* and *Bmps* act as a stimulator and inhibitor, respectively, in primary hair follicle formation as proposed in our and other previous reports (Headon and Overbeek, 1999; Mou et al., 2006; Pummila et al., 2007). *BMPs* have been extensively studied in early and late development as well as during cycling growth of hair follicles (Botchkarev et al., 2002; Botchkarev and Sharov, 2004; Guha et al., 2004; Plikus et al., 2008). Application of BMP or its antagonist, NOGGIN in skin cultures abolished the occurrence of hair placodes or generated normal hair placodes with increased density, respectively (Mou et al., 2006). These results together with other genetically modified mouse models revealed the general repressive roles of BMP signals in hair follicle induction and development. The observation of significant upregulation of the dermal condensation markers, *BMP3, BMP4*, and *BMP7* at stage 1 in our data, implied that *BMPs* also contribute to the formation of primary wool follicle dermal condensation (Takahashi and Ikeda, 1996). The similar expression patterns of *EDAR, BMP4*, and *BMP7* in our data suggest that the balance of activator and inhibitor functioned in murine primary hair follicle induction may be conserved in primary wool follicle induction (Headon and Overbeek, 1999; Mou et al., 2006; Pummila et al., 2007).

Though the mechanisms underlying murine hair follicle induction have been well studied, lncRNAs functioned in hair follicle and skin development remain largely unclear. We identified 62 differentially expressed lncRNAs and predicted their potential targets which partially overlapped with differentially expressed mRNAs and were highly enriched in pathways including WNT, TGF-β, Hedgehog, PI3K-Akt, focal adhesion, and ECM-receptor interaction. These pathways were found to be important in hair and feather follicle morphogenesis and skin development (Jung et al., 1998; Noramly and Morgan, 1998; Huelsken et al., 2001; Andl et al., 2002; Pummila et al., 2007; Michon et al., 2008; Xu et al., 2013; Zhu et al., 2013; Pan et al., 2015). Of these predicted targets, *EDARADD, FGFR2, HPSE, LRP4, SHH, SOSTDC1, TFAP2C*, and *TP63* were differentially expressed during induction of primary wool follicles and were found to play important roles in hair follicle development and morphogenesis (**Table 3**; Sutton and van Bokhoven, 1993; Li et al., 2001; Thesleff and Mikkola, 2002; Wang et al., 2008; Augusto et al., 2011; Cui et al., 2011; Narhi et al., 2012; Ahn et al., 2013; Mukhopadhyay et al., 2013).

In addition to potential marker genes of wool placode and dermal condensation discussed above, a cascade of genes related to epidermal differentiation and homeostasis were grouped, indicating the specification of granular and spinous layers of the epidermis in sheep skin. This is consistent with the anatomical changes between these two stages. When primary wool follicles formed at stage 1, the epidermis thickened dramatically from one or two homogenous layers to differentiated and stratified layers. *TP63*, a homologue of tumor suppressor *P53*, is a key regulator of epidermal development, as *Tp63* deficient mice showed blocked epidermal proliferation and stratification and no hair follicle formation in early embryonic stages (Mills et al., 1999). Upregulation of *TP63* in stage 1 suggests its important role in the embryonic epidermal development of sheep skin. Elevated expression of three type II keratins, *KRT1, KRT4*, and *KRT10*, were also highly correlated with epidermal differentiation and stratification at stage 1 of wool placode skin samples. Of these, *KRT1* and *KRT10* are two specific keratins enriched in differentiated and stratified spinous and granular layers of the skin epidermis. One type I keratin, *KRT15*, which was enriched in stage 1 samples and potentially regulated by XLOC_1807221, is mostly expressed in the hair placode and epidermis of murine E14.5 skin. Another keratin, *KRT14*, which marks the epidermal basal layer, showed elevated expression, possibly regulated by XLOC_1253177. A group of genes is involved in the adhesion of the basal epidermal sheet to the basement membrane including those encoding three laminins, three integrins, and one collagen (*Col17a1*) (McGrath et al., 1995). Two collagens, *COL5A3* and *COL2A1*, showed decreased expression, indicating their potential roles in development of the fetal sheep skin dermis. This interaction network provides a complex picture of wool follicle initiation driven by crosstalk between mRNAs and lncRNAs and strongly supports the view that these differentially expressed lncRNAs and mRNAs play important roles in sheep primary wool follicle and skin development. Additional studies are needed to determine the precise function of this small group of lncRNAs and mRNAs in wool follicle induction.

This investigation delineated global gene expression changes in mRNAs and lncRNAs during primary wool follicle induction. The fundamental knowledge of early development of primary wool follicles established in this report not only improve the understanding of wool follicle and skin development and potential refinement of wool quality, but also facilitates use of wool follicles as an additional system to assist human skin health and care. The wool developed from primary wool follicles in coarse and fine wool sheep displays very large differences in fiber thickness. The former is long and thick, whereas the latter is short and thin. It would be interesting to further elucidate the mechanisms underlying the differences between coarse and fine wool and contribute to selective breeding of wool sheep, particularly for fiber thickness.

## Author Contributions

CM designed the experiments and wrote the manuscript. YN wrote the manuscript, analyzed the data, and performed the qPCR and *in situ* validation. WC, XZ, XL, SL, and MY were involved in sample collection and staining. ZL, YH, HQ, QQ, QP, and DC were involved in sample collection and processing.

## Conflict of Interest Statement

The authors declare that the research was conducted in the absence of any commercial or financial relationships that could be construed as a potential conflict of interest. The reviewer ARAM and handling Editor declared their shared affiliation.

## Footnotes

1 http://string-db.org/

## Supplementary Material

FIGURE S1 Negative controls for *in situ* hybridization are presented applying with sense riboprobes of either XLOC_297809 **(A)** or XLOC_764219 **(B)** at stage 1 of primary wool follicle induction. In the negative controls, no hybridized signal was detected in sense riboprobes of either XLOC_297809 or XLOC_764219. Skin sections at stage 1 of primary wool follicle development in sheep fetus are shown at 400× (scale bars represent 20 μm). Epi, epidermis, Der, dermis, Pc, placode, Dc, dermal condensation.

TABLE S1 Differentially expressed transcripts between stage 0 and stage 1 of primary wool follicle induction in carpet wool sheep fetal skin.

TABLE S2 Annotation of targeted genes of differentially expressed lncRNAs.

TABLE S3 Significantly enriched GO terms of differentially expressed lncRNA target genes.

TABLE S4 Significantly enriched GO terms of differentially expressed mRNAs.

TABLE S5 Significantly enriched KEGG pathways of differentially expressed lncRNA target genes related to hair follicle and skin development.

TABLE S6 Significantly enriched KEGG pathways of differentially expressed mRNAs.
