# Supplementary figures and images for: Transcriptome Reveals Long Non-coding RNAs and mRNAs Involved in Primary Wool Follicle Induction in Carpet Sheep Fetal Skin
Source: Front Physiol. 2018 May 15;9:446. doi: 10.3389/fphys.2018.00446 (PMC5968378; doi:10.3389/fphys.2018.00446)

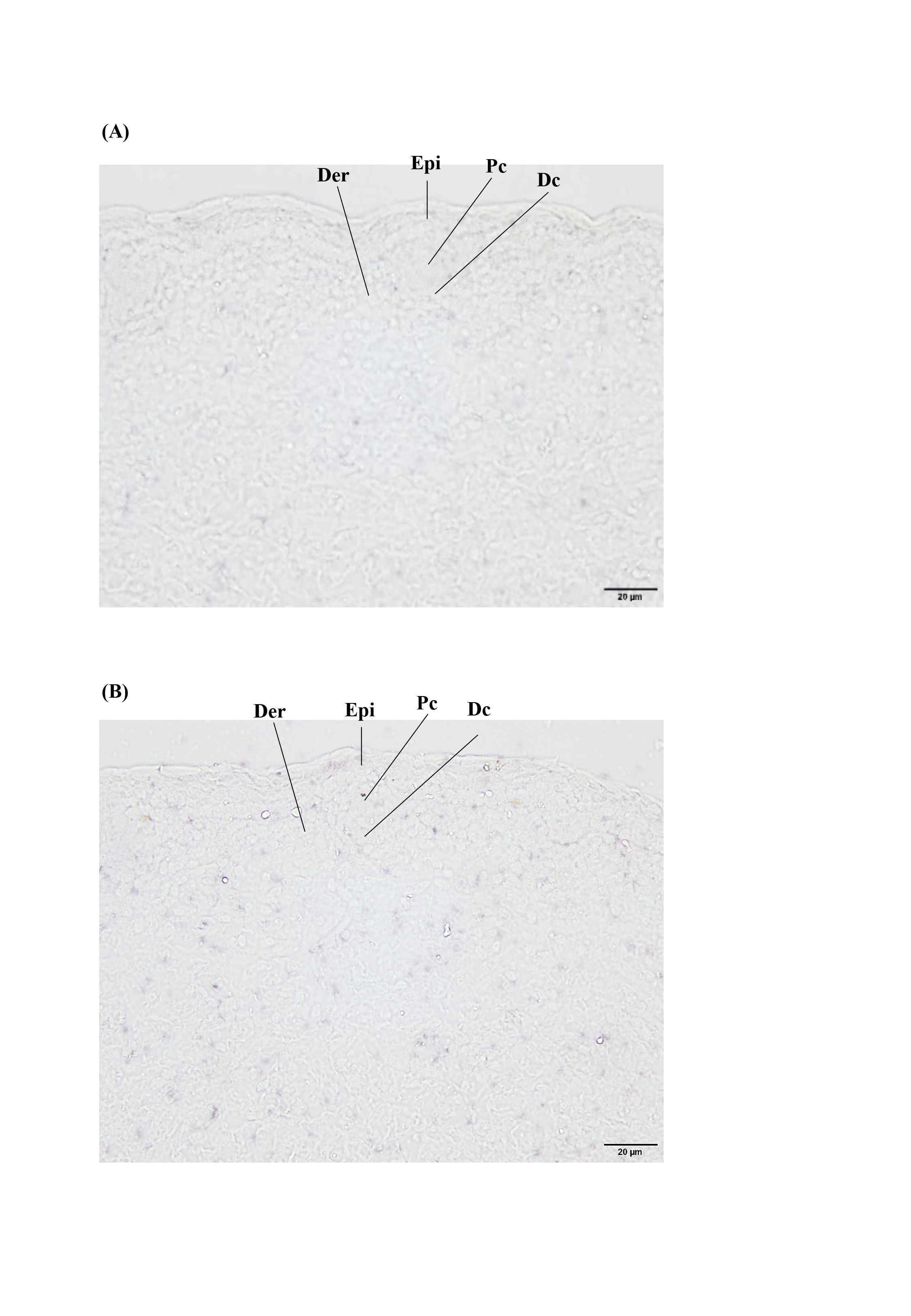

Supplement: FIGURE S1 — Negative controls for in situ hybridization are presented applying with sense riboprobes of either XLOC_297809 (A) or XLOC_764219 (B) at stage 1 of primary wool follicle induction. In the negative controls, no hybridized signal was detected in sense riboprobes of either XLOC_297809 or XLOC_764219. Skin sections at stage 1 of primary wool follicle development in sheep fetus are shown at 400× (scale bars represent 20 μm). Epi, epidermis, Der, dermis, Pc, placode, Dc, dermal condensation. [file Image_1.TIF]
